# The Effect of Poppy Oil on Egg Production and Calcium Metabolism in Japanese Quail

**DOI:** 10.3390/ani15091348

**Published:** 2025-05-07

**Authors:** Csaba Szabó, Xénia Ozsváth, Brigitta Csernus, Gabriella Gulyás, Márta Horváth, Levente Czeglédi, János Oláh, Nafiatur Rizqoh, Gabriele Achille, János Posta

**Affiliations:** 1 Department of Animal Nutrition and Physiology, Faculty of Agricultural and Food Sciences and Environmental Management, University of Debrecen, 4032 Debrecen, Hungary; mhorvath@agr.unideb.hu; 2 Department of Animal Husbandry, Faculty of Agricultural and Food Sciences and Environmental Management, University of Debrecen, 4032 Debrecen, Hungary; xenia.ozsvath@gmail.com (X.O.); gulyas@agr.unideb.hu (G.G.); czegledi@agr.unideb.hu (L.C.); postaj@agr.unideb.hu (J.P.); 3 Department of Evolutionary Zoology and Human Biology, Faculty of Science and Technology, University of Debrecen, 4032 Debrecen, Hungary; csernusb@science.unideb.hu; 4 Kismacs Experimental Station of Animal Husbandry, Institute for Agricultural Research and Educational Farm, University of Debrecen, 4032 Debrecen, Hungary; olahja@agr.unideb.hu; 5 Department of Animal Science and Biotechnology, Kyungpook National University, Sangju 37224, Republic of Korea; nafiatur.rizqoh279@gmail.com; 6 Laboratory of Protistology and Biology Education, University of Macerata, 62100 Macerata, Italy; g.achille@unimc.it

**Keywords:** poppy oil, egg production, egg quality, calcium, Japanese quail

## Abstract

Consumers tend to favor natural dietary supplements to improve their health. However, vendors rarely cite scientific evidence to justify these claims. In the case of poppy oil, it is often mentioned as having a positive effect on Ca metabolism-related disorders, but no proof has been provided. Therefore, the aim of our trial was to test the possible effect of poppy oil on calcium metabolism using Japanese quail layers as a model animal. A total of 120 four-week-old quail were divided into three dietary treatments (four cages per treatment, with ten birds in each cage): a control group (using sunflower oil as the energy source in the diet), 0.5%, and 1% poppy oil supplementation (replacing sunflower oil). Egg production, eggshell thickness, eggshell strength, and egg yolk color were investigated. Ca retention was determined using the acid-insoluble ash method. At the end of the experiment, two birds per cage were sacrificed, and uterus and jejunum samples were collected for gene expression analyses. Poppy oil supplementation improved egg production in terms of intensity, egg weight, and eggmass production. The thickness and eggshell strength decreased when 1% poppy oil was fed to the animals, while Ca retention improved. Poppy oil supplementation increased the expression of Ca transporter genes (*CALB1*, *SLC8A1*, and *SLC26A9*) in the uterus and *ITPR1* in the jejunum. Our results indicate a possible effect of poppy oil on Ca metabolism. Further studies are needed to identify the active compound and to understand the mode of action.

## Simple Summary

Non-opium poppy varieties are widely cultivated in Europe for food. The seed has a high oil content, and poppy oil is produced mainly with cold-press techniques to preserve bioactive compounds. These bioactive compounds have antioxidant and anti-inflammatory properties and improve brain function and digestion. Some vendors claim that poppy oil has a positive effect on Ca metabolism, and hence on disorders caused by the malabsorption of Ca. However, they do not provide research evidence, and we hardly found any articles dealing with such an effect. Egg-laying birds have a very intensive Ca metabolism, making them a good choice to test the effect of feed supplements. Therefore, the aim of our study was to test the effect of poppy oil on egg production, Ca retention, and Ca metabolism-related genes in a layer Japanese quail. Our results indicate that poppy oil has a positive effect on egg production and egg quality, Ca retention, and Ca transport genes in the uterus. Further studies are required to confirm our results on other animal models.

## 1. Introduction

Oils derived from plant sources, mostly from seeds, have garnered increased interest due to their beneficial compounds that have preventive and therapeutic properties, making them functional foods [1]. Dietary lipids as supplements can have a positive effect on bone mineralization [2]. Poppy (*Papaver somniferum*) is a traditional crop, grown for food and pharmaceutical purposes, mostly for opium [3]. Poppy contains alkaloids such as morphine, codeine, noscapine, papaverine, especially in the capsule [4], and physiologically active chemicals like flavonoids, phenols, sterols, and antioxidants [5]. Poppy seed oil is rich in polyunsaturated fatty acids (PUFAs); therefore, it can increase the accumulation of essential fatty acids (FAs) in the liver without negative effects if taken regularly [6]. Despite the traditional use in human medicine and food, only a few animal studies are available. Dietary poppy seed oil in Japanese quail had no effects on laying performance, reproduction, and egg quality; however, it reduced the saturated FA and increased the unsaturated FA content in the egg yolk [7]. Feed conversion ratio (FCR), eggshell thickness, and blood high-density lipoprotein (HDL) values were significantly improved when 2.5% of poppy seed oil was supplemented in the diet of laying hens [8]. When poppy seed meal was supplemented at 5%, 15%, and 25% to the quail diet, the egg production and the feed consumption increased, but the hatchability decreased [9]. The 0.5% supplementation of poppy oil decreased serum malondyaldehide (MDA) concentrations and increased glutathione (GSH) and vitamin A levels. Therefore, it can be used as a supplement to improve antioxidant status [10].

Numerous companies/traders offer cold-pressed poppy oil, and one of the often claimed effects is that it improves bone health and even reverses osteoporosis. However, this claim has not been supported by scientific evidence, and even recently published papers have not mentioned such an effect [5,11]. Japanese quails start to lay eggs around 5–6 weeks of age, and after a rapid increase, the peak production occurs between 15 and 22 weeks of age, with egg production intensity above 90%. This period is especially interesting due to the high rate of shell calcification and rapid bone turnover. Therefore, the aim of our trial was to investigate the effect of cold-pressed poppy oil supplementation on Ca retention and Ca transporters expression in Japanese quails during intensive egg production.

## 2. Materials and Methods

### 2.1. Animal Care

The experiment was performed in accordance with the EU Directive “Legislation for the protection of animals used for scientific purposes” and after approval by the Ethical Committee for animal use of the University of Debrecen, Hungary (Protocol No. 5/2021/DEMAB).

### 2.2. Animals

A total of 120 four-week-old Japanese quail (*Coturnix japonica*) chicks were purchased from a commercial quail breeder (Budai Fürjészet, Dunavecse, Hungary) and housed in quail layer cages in Kismacs Animal Husbandry Experimental Station of the University of Debrecen, Farm and Regional Research Institute (Debrecen, Hungary) in a group of ten birds. The birds had a 7-day (5th week of age) acclimation period on ad libitum access to control feed and water. The experimental room was maintained at a temperature of 18 ± 3 °C and 60–75% relative humidity. The photoperiod was fixed at 16:8 L:D daily cycle and regulated using an LED Lighting Dimming System. After the one-week adaptation, the cages were allocated to the dietary treatments (four cages per treatment) on a randomized block (level of cages) design basis. The trial lasted until the animals were 13 weeks old (for 8 weeks).

### 2.3. Dietary Treatments

Three dietary treatments were formulated (four cages per treatment): a corn–wheat–soybean-based control feed according to the authors of [12], and 0%, 0.5%, or 1% cold-pressed poppy oil (Solio Ltd., Fadd, Hungary), substituting sunflower oil in the control feed. The composition and nutrient content of the experimental diets are presented in Table 1. An amount of 0.5% of laboratory SiO_2_ was included in the diets to increase the acid-insoluble ash content in order to improve the precision of marker-based retention calculation. Feed and water were available without restriction.

### 2.4. Measurements and Samplings

Oil (sunflower and poppy oil) used in the trial was sampled before feed mixing for fatty acid, Ca, and P analyses (Table 2). Feed samples were collected after preparing the experimental diets. Feed intake was measured on a weekly basis for each cage. The number of eggs produced was recorded for each cage over the weeks. The weight of the eggs was measured for each piece, and the egg mass produced per cage per week, along with production intensity (the number of eggs produced during the week divided by the number of hen-days), were calculated. From the 9th to the 13th week of age, the average weight of the eggs for each cage was calculated after each week, and twelve eggs with average weight (three per cage) were selected for further analyses. The egg’s breaking strength was analyzed by a TA.XT plus texture analyzer (Stable Micro Systems, Surrey, UK) using a round 3 mm diameter flat head on the narrow end of the eggs. After this measurement was taken, the egg yolk and eggshell were separated. The color of the yolk was determined according to the YolkFan^TM^ (dsm-firmenich, Maastricht, The Netherlands) by the same operator. The weight and thickness (using a digital micrometer) of the eggshell were determined after cleaning the egg white remains with a paper towel. On the last day of the experiment, excreta samples were collected from the collection trays in each cage. The samples were stored at −18 °C until further analysis. At the end of the experiment, two randomly selected birds from each cage (eight per treatment) were euthanized by cervical dislocation. After opening the abdomen, samples of the uterus and 2 cm of jejunum were immediately collected for gene expression analyses. The jejunum was thoroughly washed in PBS solution; then, tissues were snap-frozen in liquid nitrogen and stored at −80 °C until further analysis.

### 2.5. Feed and Excreta Analyses

Feed and excreta samples were analyzed for dry matter (ISO 6496) [13], crude ash (ISO 5984) [14], acid-insoluble ash (ISO 5984) [14], calcium, and phosphorus. Elementary analysis was carried out after 1–2 g of samples were digested in a block digester (LABOR MIM, Budapest, Hungary) with 10 mL cc. nitric acid at 60 °C for 30 min and 3 mL of 30% hydrogen peroxide (Sigma-Aldrich, Merck KGaA, Darmstadt, Germany) for 90 min at 120 °C. The digested samples were filled to 50 mL with distilled water and filtered through MN640W (155 mm) filter paper. Analysis was carried out using the ICP-OES technique (Perkin-Elmer, Optima 3300 DV, Waltham, MA, USA).

### 2.6. Calculation of Ca Retention

The retention of calcium was calculated using the following equation (after [15]):

$$Retention\left(\%\right)=100-[(\frac{AIAd}{AIAe}\times \frac{Me}{Md})\times 100]$$

where AIAd represents acid insoluble ash content in the diet, AIAe represents excreta acid insoluble ash content, Me represents excreta Ca content, and Md represents diet Ca content (all data in g/kg DM).

### 2.7. RNA Isolation, Reverse Transcription, and Quantitative Real-Time PCR (qPCR) Assays

Total RNA was extracted from the uterus and jejunum using peqGOLD Total RNA Kit (Geldenaaksebaan, Leuven, Belgium) according to the manufacturer’s protocol, including a DNA digestion step. The concentration and purity of the RNA were quantified using a NanoDrop ND-1000 spectrophotometer (Thermo Fisher Scientific, Waltham, MA, USA). RNA integrity was checked using 1.5% agarose gel electrophoresis. A total of 600 ng of the isolated RNA was reverse-transcribed into cDNA using qScript cDNA Supermix (Quantabio, Cummings Center, Beverly, MA, USA) in a 20 µL final volume containing cDNA Supermix (5×), RNA template, and nuclease-free water. The conditions consisted of incubation at 25 °C for 5 min, reverse transcription at 42 °C for 30 min, and reverse transcriptase denaturation at 85 °C for 5 min. cDNA samples were diluted 10-fold and stored at −20 °C. Intron-spanning forward and reverse primers for quail *ATP2A2*, *ATP2B1*, *ITPR1*, *SLC26A9*, *SLC8A1*, *CALB1*, and *RPL7* were designed by Oligo 7 software (version number 7.6) (DBA Oligo Inc., Colorado Springs, CO, USA) (Table 3) and checked for target identity using National Center for Biotechnology Information (NCBI) Primer Blast web based tool (https://www.ncbi.nlm.nih.gov/tools/primer-blast/, accessed on 18 June 2024). Primer specificities were tested using melt curve analysis after each run. Quantitative PCR was performed using the LightCycler 480 Instrument II (Roche Life Science, Penzberg, Germany), and reactions were run in triplicate using 384-well plates (4titude, Surrey, UK). Each reaction included a 3 ng cDNA template, 2× Xceed qPCR SG Hi-ROX Mix (Institute of Applied Biotechnologies, Prague, Czech Republic), 200 nM of each primer, and deionized water in a 10 μL final volume. No template controls were included for each primer. Real-time PCR conditions were as follows: initial denaturation at 95 °C for 2 min, 40 cycles of denaturation at 95 °C for 5 s and annealing/extension at 60 °C for 30 s. Among the most frequently used reference genes in quail gene expression studies (*ACTB*, *GAPDH*, *RPL19*, and *RPS8*), the most stable reference gene was selected by the ΔCT, Best Keeper, and NormFinder algorithms. In both tissues, *GAPDH* was considered the most stable reference gene for normalization. Primers of candidate reference genes were published by Carvalho et al. [16]. The results were generated using the Pfaffl method [17] by normalizing the expression of the target gene to the housekeeping gene. The results were determined as fold changes in the expression of the target genes in the experimental groups compared with the control group.

### 2.8. Statistical Analyses

The production and egg parameters data were evaluated by one-way analyses of variance using SAS on Demand for Academics (v. 3.81, SAS Institute Inc., Cary, NC, USA). Gene expression results were evaluated and visualized by GraphPad Prism 8.4.3 (GraphPad Software Inc., San Diego, CA, USA) with one-way ANOVA. The Shapiro–Wilk test was utilized to assess the normality of the data, while Bartlett’s test was employed to verify the equality of variances. To analyze the effect of poppy oil on calcium metabolism and ion transport genes, a one-way analysis of variance was performed, with Tukey’s test used for mean comparisons across treatments. If the data followed a lognormal distribution, Dunn’s test was applied, and Tamhane’s T2 test was used when variances were unequal. Differences among treatments were considered significant at *p* < 0.05.

## 3. Results

**Figure 1:**
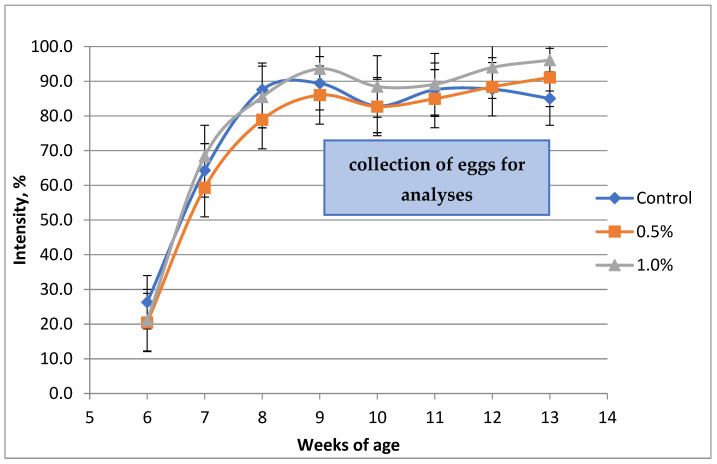
Changes in egg production intensity (%) of Japanese quails during the trial (n = 4/treatment, data presented as mean ± sd). Blue line: control group; orange line: 0.5% poppy oil addition on the expense of sunflower oil; gray line: 1.0% poppy oil addition on the expense of sunflower oil.

Egg production of the quails started on the sixth week of age, between 20 and 30% of intensity (Figure 1). Peak production was reached by the ninth week of age, around 90%. Therefore, treatment effects on measurements performed on the eggs were evaluated between the 9–13 weeks of age.

The 1% poppy oil supplementation significantly improved the egg production both in terms of quantity and intensity (Table 4) compared to the control. Egg weight increased significantly with 0.5% supplementation. As a result of these differences, egg mass production was increased by both poppy oil treatment (*p* < 0.05). The dietary treatments had no influence on feed conversion and retention of Ca.

Eggs selected for the break test had the same weight and similar eggshell weight (Table 4). The 1% poppy oil supplementation resulted in a significantly (*p* < 0.05) lower eggshell thickness, which also showed a tendency towards the eggshell strength as well. Poppy oil supplementation decreased the color score of quail eggs (*p* < 0.05).

**Figure 2:**
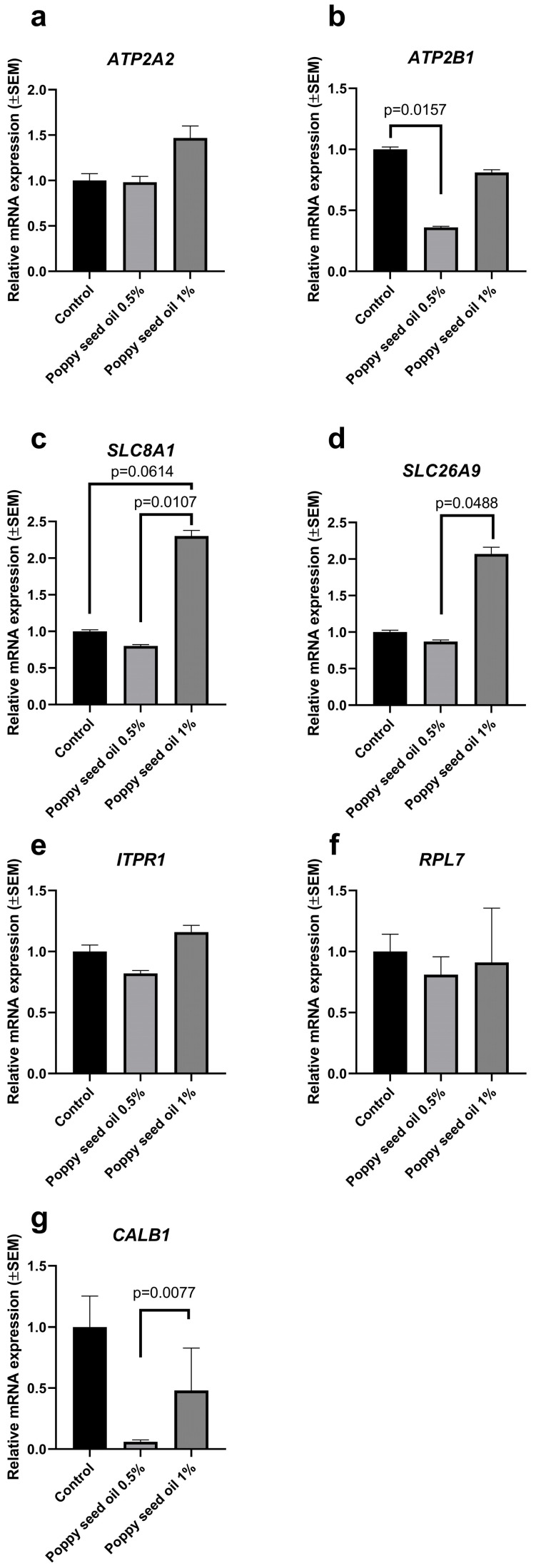
Relative ATPase sarcoplasmic/endoplasmic reticulum Ca^2+^ transporting 2 (**a**), ATPase plasma membrane Ca^2+^ transporting 1 (**b**), solute carrier family 8 member A1 (**c**), solute carrier family 26 member 9 (**d**), inositol 1,4,5-trisphosphate receptor type 1 (**e**), ribosomal protein L7 (**f**), and calbindin 1 (**g**) mRNA expressions in uterus fed basal diet and diet supplemented with 0.5% and 1% poppy oil (n = 8 birds/treatment). Data represent means ± standard errors of the mean.

In the uterus, poppy oil supplementation at 1% increased the gene expression level of solute carrier family 8 member A1 (*SLC8A1*) compared to the 0.5% group (*p* = 0.0107) and tended to increase (*p* = 0.0614) compared to the control group (Figure 2). Poppy oil at 1% further elevated the mRNA expression of solute carrier family 26 member A9 (*SLC26A9*) compared to the 0.5%-fed group. The poppy oil supplementation at 1% also elevated the gene expression level of calbindin 1 (*CALB1*) compared to the 0.5%-fed birds. Poppy oil at 1% decreased (*p* = 0.0157) the relative ATPase plasma membrane Ca^2+^ transporting 1 (*ATP2B1*) mRNA expression in the uterus compared to the control group. The gene expression levels of ATPase sarcoplasmic/endoplasmic reticulum Ca^2+^ transporting 2 (*ATP2A2*) did not change in the uterus of any of the supplemented groups.

**Figure 3:**
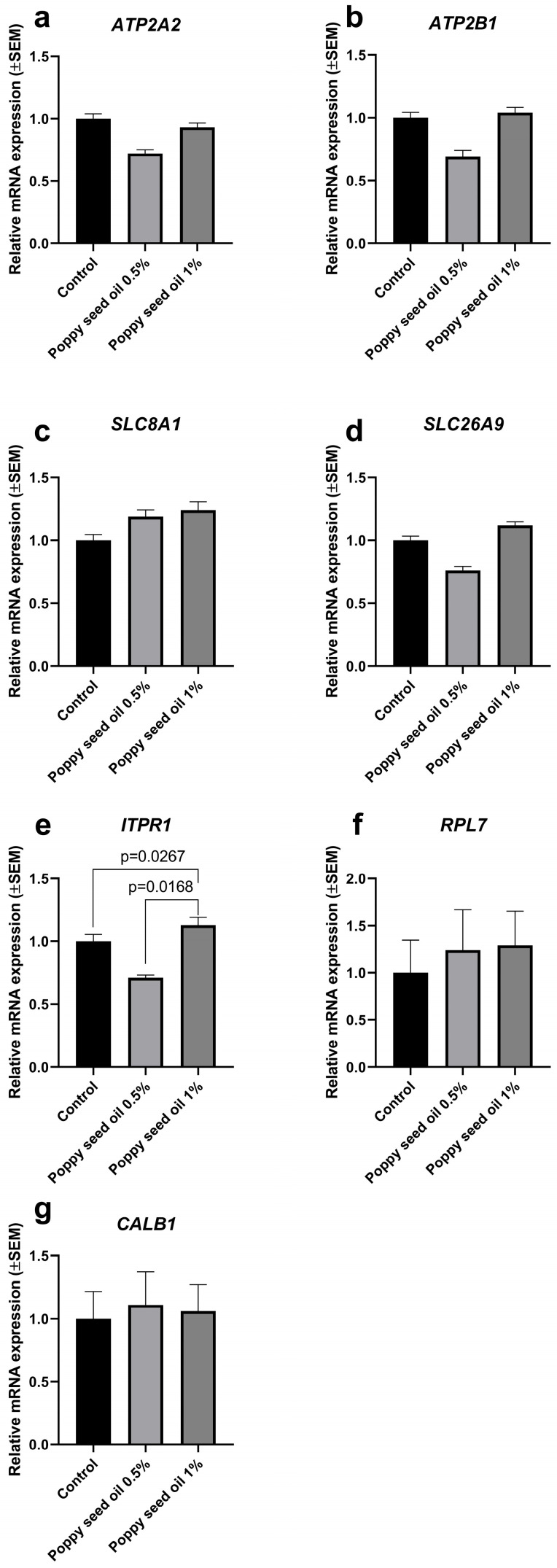
Relative ATPase sarcoplasmic/endoplasmic reticulum Ca^2+^ transporting 2 (**a**), ATPase plasma membrane Ca^2+^ transporting 1 (**b**), solute carrier family 8 member A1 (**c**), solute carrier family 26 member 9 (**d**), inositol 1,4,5-trisphosphate receptor type 1 (**e**), ribosomal protein L7 (**f**), and calbindin 1 (**g**) mRNA expressions in jejunum fed basal diet and diet supplemented with 0.5% and 1% poppy oil (n = 8 birds/treatment). Data represent means ± standard errors of the mean.

The 1% poppy oil supplementation significantly increased the expression of the inositol 1,4,5-trisphosphate receptor type 1 (*ITPR1*) gene compared to the control group (*p* = 0.0267) and to the 0.5%-supplemented group (*p* = 0.0168) (Figure 3). The gene expression levels of other genes (*ATP2A2*, *ATP2B1*, *SLC8A1*, *SLC26A9*, and *CALB1*) in the jejunum did not show significant differences.

## 4. Discussion

Similarly to others [3,18,19,20], poppy (*Papaver somniferum*) seed oil used in this trial contained linoleic acid (omega-6) and oleic acid (omega-9) as major unsaturated fatty acids, accounting for more than 85% of the fatty acids (Table 2). The most abundant saturated fatty acids are palmitic and stearic acids, representing around 10% of the fatty acids. Depending on the variety, this amount can be as much as 20% [20]. Sunflower oil contained more oleic and less linoleic fatty acid, but their sum was similar. While poppy seed contains 9–10 g/kg Ca and 9–13 g/kg P [20], the oil has a negligible amount of these minerals.

Quails started their egg production at the age of six weeks and reached about 80–90% production intensity during the 7th–8th week of age, similarly to others [21] (Figure 1). Egg production intensity was similar to what was achieved in another experiment [7]. Research results suggest that the weight of quail eggs is about 10–12 g at the peak production period [7,22], while in our experiment, we found a result above 12 g, with a small but significant increase by the 0.5% poppy oil supplementation. During the late egg production (over 40 weeks of age) phase, egg weight can be 13–14 g [23]. Feed conversion per kg egg mass was higher compared to other research results [24,25]. In the latter experiment, the birds had higher production intensity (above 90%) and the average weight of the eggs was above 13 g, despite similar age. Quite interestingly, the energy content of the diet was markedly lower (10.7 vs. 12.1 MJ metabolizable energy/kg) while the crude protein level was similar (17.5 vs. 18.3%) compared to the diet used in our experiment. The intended Ca level was the same in both cases (2.5%), but the actual values were only about 2% in our diets. These differences may explain the worse feed conversion.

Calcium retention was lower than measured by others [26,27]. Both levels of poppy oil supplementation increased the retained Ca. However, both the eggshell thickness and strength were significantly lower at 1% poppy oil inclusion. As egg production intensity and egg weight and mass produced were increased, the extra retained Ca was still insufficient to at least maintain eggshell thickness. Yolk color is an important factor that must meet consumer expectations, and its intensity is mainly dependent on the level of dietary carotenoids [28,29,30]. Carotenoids are lipophilic compounds and can easily be oxidized [31]. Oils are low in carotenoid content but are used as a carrier to prepare high carotenoid content solutions. High unsaturated fatty acid content accelerates the oxidation of carotenoids [32]. In our diets, the main source of carotenoids was corn, and it was possible that the higher linoleic acid content of poppy oil caused the detected reduction in yolk color score.

In this study, the impacts of dietary poppy oil on gene expression levels of *ATP2A2*, *ATPB1*, *CALB1*, *SLC8A1*, *SLC26A9*, *ITPR1*, and *RPL7* were measured in the uterus and jejunum of Japanese quails, as these genes are critically involved in calcium signaling and metabolism, ion transport, and protein synthesis, thereby playing vital roles in reproductive health and nutrient absorption in these tissues.

In hens, the uterus serves as the primary site for eggshell production, where mineralization occurs at the most rapid rate [33]. It has been established that multiple proteins play a role in mineralization by mediating ion transport [34]. Calcium is a crucial element in the calcification of the eggshell, continuously supplied from the bloodstream into the uterus against its concentration gradient. The transfer of calcium into the uterine fluid involves calcium channels for entry into glandular cells of the uterus. Calbindin D28k (CALB1) helps the intracellular transport, while the efflux of calcium is managed by Na^+^/Ca^2+^ (e.g., SLC8A1) and Ca^2+^/H^+^ exchangers [35,36]. Calcium levels within cells can be modulated by uptake and release mechanisms in the endoplasmic reticulum through ATP-dependent calcium pumps (e.g., ATP2A) and calcium channels (e.g., ITPRs) [34,36]. Carbonate (CO₃^2−^) constitutes another significant component of the eggshell. Within the glandular cells of the uterus, carbonic anhydrases hydrate CO_2_ to generate bicarbonate (HCO_3_^−^). Low concentrations of bicarbonate are taken up by the cell through specific transporters, including a Na^+^/HCO_3_^−^ co-transporter (e.g., SLC26A9) [34]. Bicarbonate is then exported from uterine cells into the uterine fluid through a Cl^−^/HCO_3_^−^ exchanger. The transfer of calcium and bicarbonate from the bloodstream to the uterine fluid also necessitates additional ion transport to preserve cellular homeostasis [35].

In this study, poppy oil supplementation at 1% increased the uterine gene expression level of solute carrier family 8 member A1 (*SLC8A1*) compared to the 0.5% group and tended to increase compared to the control group. Poppy oil at 1% further elevated the mRNA expression of solute carrier family 26 member A9 (*SLC26A9*) in the uterus compared to the 0.5%-fed group. The poppy oil supplementation at 1% also enhanced the gene expression level of uterine calbindin 1 (*CALB1*) compared to the 0.5%-fed birds.

In a previous study, dietary zinc supplementation did not impact the gene expression levels of Ca^2+^ transporters, including the Ca^2+^ intracellular transporter (*CALB1*), and Na^+^/Ca^2+^ exchanger (*SLC8A1*) in the eggshell gland of ducks, which have roles in the uterine calcium secretion [34,37]. However, the same dietary supplementation could elevate the mRNA expressions of HCO_3_^−^ exchangers, such as *SLC26A9*. Therefore, dietary supplementation improved calcium metabolism and deposition by modulating HCO_3_^−^ secretion [38]. *CALB1* functions as an intracellular transporter capable of moving Ca^2⁺^ across the cell, serving as both a Ca^2⁺^ sensor and a cytoplasmic Ca^2⁺^ buffer. As noted, *CALB1* expression in the uterus significantly increases during eggshell calcification and plays a vital role in the process of egg formation and positively correlates with egg quality as well [35,39].

Zhang et al. [38] also defined that dietary supplementation did not modify the gene expression level of Ca^2+^ ATPase (*ATP2A2*), although the applied feed additive ameliorated the calcium metabolism by affecting HCO_3_^−^ exchangers in the same study. In our study, poppy oil supplementation did not change the mRNA expression levels of inositol 1,4,5-trisphosphate receptor type 1 (*ITPR1*) in the uterus; however, the oil addition increased the gene expression level of jejunal *ITPR1* compared to the control group and to the 0.5%-supplemented group. The inositol 1,4,5-trisphosphate receptor is an intracellular Ca^2+^ channel that can be found in the endoplasmic reticulum, responsible for calcium ion efflux [40]. There are three isoforms (ITPR1, ITPR2, and ITPR3) that contribute to the regulation of intracellular Ca^2+^ [36]. Liu et al. [41] also reported higher mRNA expression of *ITPR1* in the ovary when capsaicin supplementation was applied, and the authors discussed that calcium-mediated signaling pathways are crucial in impacting follicle selection and maturation in laying birds [41,42]. In contrast to our results, Bahadoran et al. [35] reported lower mRNA expression of *ITPR3* in the uterus, the type 3 receptor discussed with impaired eggshell mineralization as a result of heat stress.

Additionally, ribosomal protein L7 (*RPL7*) is responsible for protein translation [43], but significant changes in the uterus or jejunum were not defined in any of the supplemented groups.

## 5. Conclusions

Poppy oil inclusion resulted in improved egg production, calcium retention, and increased levels of calcium transporter genes, which suggests that it can exert an effect on calcium metabolism. Further studies are needed to determine the active compound(s) and the mode of action.

## Figures and Tables

**Table 1:** The composition and nutrient content of the experimental diets.

	Treatment/Composition (%)		
Ingredients	Control	0.5% Poppy Oil	1.0% Poppy Oil
corn	25.79	25.79	25.79
wheat	30.00	30.00	30.00
soybean meal, 46%	30.04	30.04	30.04
sunflower oil	4.70	4.20	3.70
poppy oil	0.00	0.50	1.00
Mono calcium phosphate	1.62	1.62	1.62
limestone	5.65	5.65	5.65
salt	1.05	1.05	1.05
DL-Met	0.15	0.15	0.15
SiO_2_	0.50	0.50	0.50
premix ^a^	0.50	0.50	0.50
Total	100.0	100.0	100.0
Nutrients	Nutrient content		
crude protein, % ^b^	18.3	18.3	18.2
ME ^c^, MJ/kg	12.13	12.13	12.13
Lys, %	1.04	1.04	1.04
Met, %	0.45	0.45	0.45
Thr, %	0.74	0.74	0.74
Trp, %	0.24	0.24	0.24
Ca, % ^b^	2.03	2.14	2.23
total P, % ^b^	0.70	0.78	0.74
non phytate P, %	0.35	0.35	0.35
Na, %	0.4	0.4	0.4

^a^ The 1 kg premix provided: 1,000,000 NE vitamin A, 200,000 NE vitamin D_3_, 4900 mg/kg vitamin E, 200 mg vitamin K_3_, 150 mg vitamin B_1_, 500 mg vitamin B_2_, 1200 mg Ca-d-Pantothetane, 400 mg vitamin B_6_, 2 mg vitamin B_12_, 11 mg biotin, 2502 mg niacin, 60 mg folic acid, 300,000 mg choline cloride, 13,200 mg Zn, 1920 mg Cu, 9612 mg Fe, 13,200 mg Mn, 180 mg I, 42 mg Se, and 12 mg Co. ^b^ Analyzed values. ^c^ Metabolizable energy.

**Table 2:** Fatty acid, Ca, and P content of sunflower and poppy oil used in the trial.

Nutrient	Sunflower Oil	Poppy Oil
Myristic acid C14:0, %	0.06	0.06
Pentadecanoic acid C15:0, %	0.01	0.01
Palmitic acid C16:0, %	6.12	8.77
Palmitoleic acid C16:1, %	0.07	0.14
Margaric acid C17:0, %	0.04	0.06
Heptadecanoic acid C17:1, %	0.03	-
Stearic acid C18:0, %	3.14	2.07
Oleic acid C18:1c, %	26.41	14.13
Octadecanoic acid isomer C 18:1, %	0.61	1.23
Linoleic acid C18:2c, %	60.82	72.81
Arachidic acid C20:0, %	0.21	0.09
Eicosenoic acid C20:1, %	0.14	0.07
α-Linolenic acid C18:3n3, %	1.44	0.56
Behenic acid C22:0, %	0.69	-
Tricosanoic acid C23:0, %	0.02	-
Lignoceric acid C24:0, %	0.20	-
Ca, mg/kg	44.5	37.6
P, mg/kg	85.6	0.50

**Table 3:** Genes analyzed in the experiment.

GenBank No.	Gene Symbol	Gene Name	Forward and Reverse Primer	Product Length (bp)	Annealing Temperature (°C)
XM_015877724.2	*ATP2A2*	ATPase sarcoplasmic/endoplasmic reticulum Ca^2+^ transporting 2	F: AGCGTTCAGAGAATCAAAGCAAG	81	54.81
			R: ATCAGCAGGAACCTTGTCTCCA		56.36
XM_015277056.2	*ATP2B1*	ATPase plasma membrane Ca^2+^ transporting 1	F: CTCGGCGCTGCCCGGTG	103	61.38
			R: CCATGACGAGCTGTGTTCCCCAA		60.11
XM_032447330.1	*ITPR1*	inositol 1,4,5-trisphosphate receptor type 1	F: ACAGCCAGAAGCAGGTGACCTTA	118	58.74
			R: CGGCTTTGCTGCTTTCCAGAACT		59.25
XM_015855985.2	*CALB1*	calbindin 1	F: ACGACTCCGACGGCAATGGGTA	120	60.93
			R: CCACAAAGGCTTTCATTTCGGGT		57.12
XM_015885285.2	*SLC26A9*	solute carrier family 26 member 9	F: GCTCTTCTCTCCGTGCCACCT	106	59.15
			R: CGTTCGATGGCATAGCGGGGTC		60.78
XM_032443672.1	*SLC8A1*	solute carrier family 8 member A1	F: CACCTGTGGGGAGCTGGAGT	113	58.67
			R: CCCCGATCTCTAGGTAGAAGGTCTT		57.46
XM_015855784.2	*RPL7*	ribosomal protein L7	F: ACTTTGTGGAGGGTGGAGATGCT	96	58.77
			R: AAACTGCAGCTGGGCATCTGA		57.62
XM_015873412.2	*GAPDH*	glyceraldehyde-3-phosphate dehydrogenase	F: TCTCTGTTGTTGACCTGACCTG	155	54.90
			R: ATGGCTGTCACCATTGAAGTC		53.56

**Table 4:** The effect of poppy oil supplementation on the production parameters (n = 4/treatment) and egg measurements between the 9th and 13th week of age.

	Poppy Oil Inclusion			RMSE	*p*
	Control	0.5%	1.0%		
Number of eggs produced per week	57.2 ^b^	62.3 ^ab^	66.3 ^a^	6.76	0.0004
Production intensity, %	86.5 ^b^	86.6 ^b^	92.2 ^a^	6.78	0.0133
Egg weight, g	12.29 ^b^	12.59 ^a^	12.35 ^ab^	0.34	0.0160
Egg mass per week, g	703.4 ^b^	783.6 ^a^	818.1 ^a^	89.1	0.0005
Feed conversion, g/egg	39.8	40.1	38.1	3.50	0.1674
Feed conversion kg/kg egg mass	3.24	3.18	3.09	0.25	0.1645
Ca retention, %	9.45 ^b^	18.90 ^a^	21.62 ^a^	2.49	0.0114
Traits of eggs used for strength analyses					
Egg weight, g	12.7	12.7	12.7	0.53	0.9842
Eggshell weight, g	1.17	1.20	1.17	0.07	0.3318
Eggshell thickness, µm	268.3 ^ab^	268.5 ^a^	255.7 ^b^	16.7	0.0277
Eggshell strength, N	14.1	14.7	13.4	1.74	0.0834
Yolk color ^c^	5.36 ^a^	4.67 ^b^	4.51 ^b^	0.52	0.0001

RMSE: root mean square error; ^a,b^ means in a row with common superscript do not differ (*p* > 0.05). ^c^ Color score was determined with YolkFan^TM^.

## Data Availability

Data are available upon reasonable request from the corresponding author.
